# First- and Second-Generation Psychological Theories of Suicidal Behaviour

**DOI:** 10.3390/bs14080710

**Published:** 2024-08-14

**Authors:** Elif Yöyen, Merve Keleş

**Affiliations:** 1 Department of Psychology, Faculty of Humanities and Social Sciences, Sakarya University, Sakarya 54050, Türkiye; 2 Department of Educational Psychology, College of Education, Texas Tech University, Lubbock, TX 79416, USA

**Keywords:** suicide, first-generation psychological theories, second-generation psychological theories

## Abstract

Suicidal behaviour is defined as taking actions with the intention of killing oneself and thinking of performing these actions. Suicide is a serious public health problem with complex biological, social and psychological risk factors and a multidimensional clinical appearance, occurring all over the world and ranking among the leading causes of death. In this study, psychological approaches explaining suicide were evaluated under the headings of first-generation and second-generation suicide theories, the different aspects of these theories were put forward, and previously published studies and recent evidence were reviewed. A literature review on the theoretical foundations of suicide is presented. First-generation suicide theories were developed before the 2000s and second-generation suicide theories were developed after the 2000s. While Psychodynamic Theory, Social Learning Theory, Hopelessness Theory, Shneidman’s Suicide Theory and Escape Theory are included under the title of first-generation suicide theories, Interpersonal Psychological Suicide Theory, Three Stage Suicide Theory, Complementary Motivational-Demotivational Theory and Variable Predisposition Theory are examined as second-generation suicide theories. The approaches of the theories provide important evidence in understanding suicidal behaviour and recognising various risk factors in the transition from suicidal ideation to suicidal action. Controlling the risk factors may contribute both in terms of preventive community mental health and in the development of health policies.

## 1. Introduction

Since suicide is a very complex and subjective concept, it is difficult to make a definition that is accepted by everyone, but it is necessary to make a common definition due to scientific requirements [1]. Suicidal behaviour is defined as taking actions with the intention of killing oneself or thinking of taking these actions. The concepts expressed by suicidal behaviour are death by suicide, suicide attempts and suicidal ideation [2]. According to WHO data for 2021, due to suicidal behaviour, which constitutes a serious public health threat all over the world, one person dies every 40 s and over 700,000 people die by suicide every year [3]. Research to understand suicidal behaviour has focused on identifying the risk factors that determine suicidal behaviour. The World Health Organization (WHO) has defined a five-step risk factor chain related to suicidal behaviour as “individual factors, relational factors, factors related to the living environment, social factors and factors related to the health system” [4].

Theories that try to understand the underlying causes of suicidal behaviour with biological, psychological and social approaches provide strong evidence in each field [5]. In the biological approach, genetic factors [6,7], dysfunction of the serotonergic system in the neurobiological system; HPA axis hyperactivity; noradrenergic hyperactivity; dopaminergic, glutamatergic system, and GABAergic system dysfunction; anomaly and signalling error in glial cells and microgliosis are thought to be responsible [8,9,10]. Studies have shown that there is a decrease in serotonergic activity in the brain of people with suicidal behaviour and a decrease in the level of 5-Hydroxy indole acetic acid (5-HIAA) in the spinal fluid [11]. The compound 5-HIAA was found to be associated with impulsivity [12]. Lidberg and colleagues examined 35 people arrested for murder and found that 1 in 3 of these people attempted suicide and their 5-HIAA levels were lower than those who did not attempt suicide. In the same study, it was observed that 5-HIAA levels were lower in individuals with impulse control disorder [13].

In the sociological approach, it is seen that Emilie Durkheim (1897) deals with suicide from a sociological perspective. According to Durkheim, suicide occurs as a result of irregularities in the relations between the individual and society. At the same time, according to the theory, societies have social suicide rates and this rate does not change unless there is an extraordinary situation or social change in the society. In this case, while the integration of the individual and society leads to a decrease in the number of suicides, isolation from society leads to an increase in the number of suicides. Durkheim, explains his theory through two main dimensions: social integration and social regulation. The first of these, social integration, refers to the individual’s acceptance by the society and feeling as a part of the community, while social regulation means the regulation of the individual’s irrational wishes and desires by social norms and rules. Durkheim defined four types of suicide that may occur according to the levels of social integration and social regulation: Selfish (egoistic) Suicide: it occurs as a result of the weakening of the ties between the individual and society and the inability of the individual and society to integrate. Altruistic Suicide: in societies where social integration is very high, it is a type of suicide by the individual due to social and moral values. Anomic Suicides: according to Durkheim, positive or negative changes in social life lead to irregularities and the balance between the individual and social life is disturbed. When serious changes are made in the social structure, people may turn to suicide because of the disrupted balance. In this type of suicide, which is especially seen in societies where social regulation and control are low, the inability of the individual to regulate his/her behaviour and the lack of guidance leads to a kind of depression. In cases of economic downturns or upturns, social crises and changes, suicides of deregulation can be seen more frequently. In other words, it is a type of suicide that comes to the fore in rapid social/cultural/economic change processes, when the norms that bind the person to the society lose their function and the person cannot adapt to the new structure. Fatalistic Suicides: it is a type of suicide seen in societies with too much social regulation. The existence of too many and strict rules pushes the person to look for a way of escape and the person may take his/her own life as a salvation. Fatal suicide is a phenomenon arising from the excesses of regulations. Durkheim mentions many extreme cases that lead to this fourth type: “suicides of persons whose future is cruelly restrained, whose passions are restrained by oppressive discipline”, of the “very young husband”, of the “childless married woman”, of the “slave suicide”, in short, suicides that “may be attributed to the excesses of material or spiritual despotism.” In this type of suicide, the life of the individual has been determined by a normative structure and the individual has completely lost hope of escaping from this situation in the future. The individual sees suicide as the only salvation. There are rules in such societies, but they are not fair [14].

Although many risk factors have been identified in the development of complex and multifaceted suicidal behaviour, it is still unclear how these risk factors that increase the risk of suicide work together [15]. In what context can it be so dominant for human beings, who biologically have a very strong life impulse, to end their lives? In the field of psychology, many theories have been proposed to explain the development of suicidal behaviour. First-generation psychological theories (Psychodynamic Theory, Social Learning Theory, Hopelessness Theory, Shneidman’s Suicide Theory and Escape Theory) have made a great contribution to generating interest and hypotheses about suicidal behaviour [16]. Later, since the 2000s, new, second-generation psychological theories (Interpersonal Suicide Theory, Three Stage Suicide Theory, Integrative Motivational-Demotivational Theory and Variable Predisposition Theory) have been developed to explain suicidal behaviour. These newer theories assume that the factors leading to the development of suicidal ideation are different from behavioural principles such as suicide attempts or death by suicide. In this review, “first-generation (traditional generation)” psychological theories and “second-generation (new generation)” psychological theories explaining suicidal behaviour will be evaluated.

## 2. First-Generation Psychological Theories of Suicide

### 2.1. Psychodynamic Theory

According to psychoanalytic theory, the suicide process starts with the unconscious loss of the object of love. Unlike the mourning process seen in the loss of the real love object, in the process of depression, there is an unconscious loss of the internalised love object. In this case, when the need for love and attachment to the object of love is prevented, the person starts to direct his/her feelings of anger and resentment towards the self instead of the real or imaginary object. When the individual loses interest, libido is withdrawn from the object of love, but when it cannot be invested in another object, it returns to the individual, the self. The loss decreases the self-worth of the individual. Suicidal behaviour, which is a form of self-destructive behaviour, emerges when an individual experiencing feelings of abandonment and meaninglessness towards life turns to the self with feelings of hostility such as revenge and destruction [17].

According to the Object Relations Theory, which emphasises the importance of the relationship established with the caregiver in the formation of personality, the infant constructs and internalises relationships in his/her mental world. In this process, the object is categorised as good or bad depending on whether it reacts according to the baby’s needs. According to Melanie Klein, suicide stems from unconscious dynamics developed to remove internalised bad objects [1].

Karl Menninger (1938) underlines three basic motives that are fundamental in suicide. These motives are the desire to kill, the desire to be killed and the desire to die. The desire to kill is formed by feelings of anger and resentment. When the individual directs this feeling of anger towards the self, the desire to be killed emerges and these emotions directed towards the self reveal the desire to die [1]. Ericson, on the other hand, suggested that individuals experience anxieties about the realisation of their expectations with the insufficient development of the sense of productivity and stated that the feeling of depression will occur with the increase of these anxieties [18].

### 2.2. Social Learning Theory

According to the Social Learning Theory [19], human behaviour is formed through learning. Accordingly, a child creates his/her own behavioural repertoire by observing the people they choose as role models. Lester (1987) stated that this approach affects the suicidal behaviour of people with suicidal intentions by focusing on their childhood experiences. He also stated that other factors are not insignificant in explaining suicidal behaviour, but they are not sufficient [19].

According to the Social Learning Theory, suicidal behaviour occurs as a result of learning. People who have suicidal thoughts, attempt suicide or plan suicide learn suicidal behaviour from others or perform this behaviour as a result of negative events. Cultural attitudes, accessibility of suicide tools and stressful life events may be determinants in suicide attempts [1].

### 2.3. Hopelessness Theory

Hopelessness theory was developed as a response to the limitations of Seligman’s (1972) learned helplessness theory of depression [20]. According to Seligman, when living beings believe that their behaviour has no effect on the outcome of an event or situation, they exhibit a learned helplessness response. In other words, learned helplessness is the state in which a living being remains unresponsive to situations that it cannot control through its behaviour, even when it later becomes able to control them. Helplessness, which refers to the perception of personal inability to make any changes, is caused by the inability to control the outcomes of a situation in clinical depression and related mental illnesses. Hopelessness, which refers to the perception that others are also incapable of making any changes, is called ‘hopelessness depression’, a subtype of depression [21]. According to the hopelessness theory, suicidal tendency, which is considered a process that continues from suicidal ideation to the completion of suicide, is the most important symptom of hopelessness depression [22].

In the theory of hopelessness, while negative events play a decisive role in people’s hopelessness, three different inferences (internal–stable–global causes, negative consequences, negative self-features) at the end of these events make people feel hopeless and thus increase their suicidal tendencies [22]. At the same time, Abramson emphasised the place of the ‘cognitive vulnerability’ component in the theory of hopelessness and stated that having a negative cognitive style contributes to the increase in hopelessness. Thus, it has been stated that the feeling of hopelessness occurs with the mentioned elements and that suicide and other symptoms of hopelessness depression are seen due to this.

In a study examining suicide risk through the hopelessness model as an explanation of the link between childhood maltreatment and adult suicide, Gibb et al. (2001) found that suicide risk supports the hopelessness model. In the same study, cognitive risk and hopelessness levels were found to partially mediate the relationship between emotional maltreatment in childhood and suicidal ideation [23]. In addition, in a study investigating the relationship between self-coherence, hopelessness and suicidal ideation, Cornette et al. (2009) stated that the real, ideal, future component of self-coherence contributes to hopelessness, thus increasing the level of suicidal ideation and depression. It is emphasised that this result is consistent with the hopelessness theory of suicide [24].

### 2.4. Shneidman’s Theory of Suicide

Shneidman (1998) emphasised the psychological components of suicide and described these components as the ‘trunk of suicide’ to emphasise their importance [25]. In this analogy, other components, such as the method of suicide, the content of the suicide note and its effect on those left behind, are considered branches, defective fruits and leaves [26].

Shneidman states that suicide is mainly caused by severe psychological pain, personal anguish, restlessness and suffering. ‘Psychache’, defined as psychological pain, is considered a key factor among the psychological factors in suicide. Shneidman argues that unbearable pain is caused by the inhibition of psychological needs and that all emotional states (depression, hostility, anger, apathy, guilt, shame, hopelessness, etc.) are related to suicide because they cause unbearable psychological pain [26].

Shneidman has categorised the features that he states as common points in suicide in the following 10 items. Although these are not dangerous on their own, he states that the combination of many of them will cause suicide. 1. The person focuses on the unbearable pain and how to escape from it. It is the unbearable pain that leads the person to suicide. Individuals who experience positive emotions do not tend to kill themselves. 2. The fact that the person’s psychological needs (protection, trust, friendship, success, etc.) are blocked causes the tension in suicide. As a result of the inhibition of the needs, the act of suicide is realised. 3. Suicide does not occur without purpose or reason. The purpose of suicide is to seek a solution. Death provides a solution by allowing the person to resolve the unbearable situation of isolation and discomfort. 4. Another purpose of suicide is ‘loss of consciousness’, which is seen as a way out of mental suffering. The person wishes to end all conscious life and endeavours to achieve this goal. 5. The person is devastated; they feel rejected, unsuccessful and hopeless. The feeling of loss of control is added to the feelings of inadequacy, hopelessness and loneliness, and the person becomes fearful of getting out of control. The person who has no faith in recovery prefers to die rather than lose control. 6. The person is torn between conflicting feelings, wishes and behaviour. The options for a solution are reduced. The person is stuck between life and death. 7. The person poisons the self with intense emotions and contradictory logic and perceptions. Ambivalent feelings arise and the person kills the self on the one hand and calls for help on the other. 8. The person wants to withdraw from the scene, move away, go away and disappear. The person informs his/her relatives about their suicidal intentions in various ways. In this way, the person tries to be understood and helped. 9. The person exhibits behaviour patterns that shorten his/her life. Suicide is an irreversible separation. 10. The person’s communications begin to have subconscious psychodynamic implications. Suicide is a way of solving previous problems [27].

In other words, these 10 points can be summarised as follows: The common goal of suicide is to find a solution. The common goal of suicide is the removal of consciousness. The common stimulus of suicides is unbearable psychological pain. The common stress of suicides is thwarted psychological needs. The common emotion of suicides is helplessness–hopelessness. The common mental state of suicides is one of dilemma. The common perceptual state of suicides is being trapped. The common action of suicides is exit. The common interpersonal act of suicides is the expression of intention. The common consistency of suicides is lifelong coping patterns.

### 2.5. Escape Theory

Baechler (1980), another theorist, considered suicide as a way of solving problems and adopted a rationalist perspective towards suicide [28]. Although this view is an important step in evaluating suicide as an escape, it is seen as incomplete. Thus, Baumeister (1990) presented a new version of the escape theory and saw suicide as a final step in the attempt to escape from oneself and the world. According to the theory, the processes leading to suicidal behaviour consist of six stages. In the first stage, the person believes that they cannot meet the standards set by the self or someone else and that the current situation is not at the desired level. This difference may result from unrealistically high expectations or stressful life events that the person has recently experienced. According to the theory, a person with low expectations is more likely to consider suicide as a result of a stressful life event than a person with high expectations. In the second stage, the person makes internal attributions by attributing the disappointing results to the self. They make negative accusations about the self and believe that what happened was caused by their own inadequacy. These accusations reveal feelings of worthlessness and rejection, which are common in suicide attempters [29].

In the third stage, the person experiences a high level of negative self-awareness and sees the self as inadequate, incompetent and unloved, or guilty because they fall short of the goals. High levels of perfectionism cause the person to fall behind their goals and blame the self due to high expectations from the self or their environment. In the fourth stage, negative conditions such as anxiety and depression arise due to negative self-awareness. In the fifth stage, cognitive destruction occurs in the person and the meaningfulness and integrity of the individual’s inner life decrease with the negative emotional state and the thinking skills and self-awareness weaken. The three main elements of cognitive destruction are a narrow perception of time focused on the present moment, orientation towards short-term goals, and focus on instant emotions and actions. In the sixth stage, according to the theory, there are four results that lead the person to suicide: the disappearance of internal barriers/prohibitions that prevent the person from suicide, the feeling of helplessness and passivity due to the inability to solve the problems faced by the person; the display of an artificial absence of emotion by suppressing the intense negative emotions experienced; the emergence of irrational cognitions as a result of cognitive destruction (dysfunctional attitudes, cognitive rigidity, etc.) [29].

## 3. Second-Generation Psychological Theories of Suicide

Many theorists such as Shneidman, Durkheim, and Baumeister have provided very useful information in suicide explanation, research and prevention studies. However, the theories put forward are insufficient in distinguishing between suicidal thoughts and suicidal behaviour, considering that most people with suicidal thoughts do not attempt suicide. In order to overcome this gap, studies have started to be carried out within the framework of moving from thought to action. Accordingly, different explanations, predictors and processes explain the development of suicidal ideation and the progression from ideation to potentially fatal attempts [30]. The theories developed since the early 2000s, which can be called ‘second generation’ or ‘new generation’, assume that the factors that play a role in suicidal ideation and suicide attempts or deaths due to suicide are not the same [31]. Suicide theories, which are considered within the framework of thought-to-action, provide testable and promising hypotheses in the process of progression from suicidal ideation to suicide attempts [32]. The theories that will be discussed in this context are Interpersonal Psychological Suicide Theory, Three Stages Suicide Theory, Integrative Motivational-Demotivational Theory and Variable Predisposition Theory.

### 3.1. Interpersonal Psychological Theory of Suicide

According to the Interpersonal Psychological Theory of Suicide (IPTS) proposed by Joiner (2005) [33], suicidal desire emerges with the combination of feelings of inhibited belonging and a perceived burden on others. However, the realisation of suicidal behaviour requires the individual to have acquired suicidal competence in addition to suicidal desire [34]. In this case, suicidal desire is necessary but not sufficient for a fatal suicide attempt.

Van Orden et al. (2010) described Joiner’s (2005) theory more clearly and argued that suicidal behaviour emerges through habitual and opposing processes that the individual develops when repeatedly exposed to physically painful and/or frightening experiences [35]. Painful and provocative events such as childhood maltreatment, self-starvation seen in anorexia, and non-suicidal self-harming behaviours may cause individuals to ‘acquire’ the ability to suicide by causing them to become accustomed to fear and pain. Past suicide attempts may function in a similar way [33].

Thwarted belongingness occurs in cases where the need for belonging, which is a basic psychological need of the person, is not met and social attachment is damaged (loneliness, loss of spouse, etc.). According to the theory, when the need for belonging is not met, suicidal desire develops, which can also be called passive suicidal ideation. Thwarted belongingness is considered a multidimensional structure. This structure includes the factors of loneliness and mutual care relationships, which are observable and associated with a high risk for fatal suicide attempts. The loneliness factor explains some observable risk factors in fatal suicidal behaviours. The first of these risk factors is self-report of loneliness, which means the observable expression of loneliness through self-report. The second risk factor, togetherness, refers to coming together as a result of positive collective experiences. Seasonal variability leads to an increase in loneliness with a decrease in social interaction and is seen as a risk factor in the peak of fatal suicidal behaviour, especially in the spring period. While being married, having children and having friends reduces loneliness, living alone, low social support and having a disrupted family structure (being separated from one or both biological parents) are risk factors for loneliness [35].

The second dimension of impeded belonging is defined as the absence of mutual care relationships. Mutual care relationship refers to relationships in which individuals both care for themselves and care for others. The absence of mutual care relationships is explained by the emergence of six observable risk factors for lethal suicidal behaviour: social withdrawal, solitary confinement, domestic violence, loss due to divorce or death, childhood abuse and familial conflict [35]

The perceived burden on others is defined as the feeling that one is a burden on family, friends or other close people. Being a burden on others consists of two components: the belief that the self is defective enough to create a responsibility for others and self-loathing. The self-burdening component can be expressed as “I make things worse for the people in my life”, while the self-loathing component can be expressed as “I hate myself” or “I am useless”. In the context of suicide, the perception of oneself as a burden can be seen in six ways: unemployment, homelessness, incarceration, suffering from physical illness, not being wanted/available and the belief that one is a burden on the family. Self-hatred manifests itself in three categories: low self-esteem, shame and self-blame. Self-hatred, which is the other dimension of perceived burden, is expressed by three observable risk factors. These are low self-esteem; self-blame and shame; and mental agitation [35]. Although the decrease in one’s sense of belonging to others and perceiving oneself as a burden to others leads to suicidal desire, the acquired suicidal competence must also be high for a fatal suicide attempt to occur [35,36]. In addition, hopelessness is considered a factor that increases both suicidal ideation and suicide attempts [34].

According to the theory, in order for an individual to die by suicide, they must lose some of his/her fear of suicidal behaviour. It is stated that fearlessness of death is acquired as it is very unlikely to be an innate trait. Repeated exposure to physical pain and/or fear makes it possible to engage in lethal self-injurious behaviour by increasing suicidal competence by increasing the pain threshold and decreasing the fear of death. This process is referred to as acquired suicidal competence. In the theory, childhood maltreatment, past suicide attempts, encountering/clustering with suicidal people, exposure to war and impulsivity are defined as opposing processes that are physically painful and provide habituation to fear. Childhood abuse may activate the habit of pain tolerance, which increases with the fear of self-harm. Encounters with others who have engaged in suicidal behaviour may explain clustered suicidal behaviour by activating the habituation to fear of suicidal behaviour. Exposure to war represents a direct pathway in acquired competence as it expresses fear of possible death and killing others [35].

### 3.2. The Three-Step Theory of Suicide

Klonsky and May (2010) consider the framework of moving from thought to action, inspired by Joiner’s (2005) work [33], as important for guiding suicide researchers [30]. According to Klonsky and May, the development of suicidal ideation and the transition from suicidal ideation to suicide attempt should be considered as separate processes with different explanations. Accordingly, the theory explains suicidal ideation and attempts with four factors: pain, hopelessness, commitment and suicidal capacity [36].

The first step of the theory is the development of suicidal ideation. Suicidal ideation starts to develop with ‘pain’ regardless of its source. The concept of pain here, although not obligatory, usually refers to psychological or emotional pain and can be caused by any reason (physical suffering, social isolation, disappointment, low sense of belonging, etc.). However, pain alone is not sufficient for the formation of suicidal ideation and ‘hopelessness’ should be added to it. In this case, a person will think of suicide when they experience pain in their daily life and have no hope that it will become better. In summary, the combination of pain and hopelessness constitutes suicidal ideation. Suicidal ideation will not occur when pain and hopelessness are seen alone. May and Klonsky (2013), in their comprehensive study of suicide motivations, showed that pain and hopelessness are the two most common motivations for suicide attempts [37].

The second step of lethal suicidal behaviour is referred to as commitment. This broad term usually refers to being connected to people, but it can also refer to a commitment to a job, project, role, hobby or anything else in life in which one is invested. Commitment is important to determine the degree of suicidal ideation, that is, to understand whether it is strong or moderate. If an individual experiencing pain and despair does not have a sense of commitment (or if it is less than pain), this person will have strong suicidal ideation and will have an active suicidal desire. On the other hand, if a parent experiencing pain and despair has more investment or attachment to his/her children than pain, they may have passive suicidal ideation, but this ideation will not turn into an active suicidal desire. Although the concept of interrupted commitment in the theory is similar to the concepts of thwarted belonging and burdening others expressed in the Interpersonal Suicide Theory, there are some differences between these concepts. The main role of commitment is to be protective of individuals with strong suicidal ideation due to pain and hopelessness. Disruption of commitment contributes to pain and hopelessness but is not necessary for the formation of pain, hopelessness or suicidal ideation. Other risk factors (various disorders, personality traits, temperament, experiences, etc.) identified in the theory for suicide are also important, but it is argued that they may be related to suicidal ideation through affecting pain, hopelessness and/or commitment [38].

The third stage of the theory (from ideation to attempt) is whether or not, when a person feels the desire to end their life, they are likely to act on that desire and attempt it. Therefore, even if people have a strong suicidal ideation, it is not easy for them to attempt suicide due to their biological and evolutionary tendency to avoid pain, injury and death. Klonsky and May extended the variables contributing to suicidality and analysed them in Joiner’s theory [39]. In addition to the acquired category, which corresponds to the same concept, they added the categories of predisposition and practice, suggesting three categories in total. Predisposition refers to variables such as sensitivity to pain, which is largely determined by genetics. Practicality refers to concrete factors that facilitate suicide attempts, and these factors vary. For example, it is possible to say that a person who has access to and knowledge about firearms is closer to turning suicidal thoughts into action than those who do not have access to firearms. According to the theory, one or more of the following are recommended for suicide prevention and intervention efforts. (a) Reducing pain, (b) increasing hope, (c) improving communication and (d) reducing the capacity for suicide attempts. The interventions to be applied may vary according to factors such as the environment where the treatment will be applied, the age of the person and the approach to be applied [38].

### 3.3. Integrative Motivational–Deliberative Model

Rory O’Connor provides a comprehensive explanation of the transition from suicidal ideation to suicide attempt and the formation of suicidal ideation in the model he developed in 2011 and revised in 2018 [39]. The Complementary Motivational-Volitional Model explains the development of suicide in three stages: the pre-motivational stage, the motivational stage and the volitional stage.

The pre-motivational stage consists of background risk factors and triggering situations. These are categorised under three headings: diathesis, environment and life events. Diathesis are factors that increase the risk of suicide such as biological, genetic or cognitive vulnerability or individual differences. While socio-economic inequalities and rapid social changes are referred to as environmental factors, life events refer to stressful situations that occur in any period of life [15,39].

In the second stage, the motivation stage, the focus is on the psychological processes that lead to the formation of suicidal thoughts and intentions. These are, respectively, feelings of disappointment and humiliation, feeling trapped, suicidal ideation and intention. The intermediate pathway that facilitates the transition from the stage of frustration to the stage of entrapment is ‘self-oriented factors’ (rumination, etc.). The intermediate pathway that facilitates the transition from the stage of being trapped to suicidal ideation is ‘motivational factors’ (thwarted belonging, being a burden to others, etc.). The intermediate pathway that facilitates the transition from suicidal ideation to suicide attempt and increases suicide attempts in the presence of suicidal intention is ‘volitional factors’ (impulsivity, pain tolerance, fearlessness of death, etc.) [15,39]. The last stage, the will stage, involves the realisation of suicidal behaviour. In summary, according to the theory, being disappointed and trapped are associated with suicidal ideation, and volitional variables play a greater role in suicide attempts than acquired suicidal competence. Although the theory is new, it is supported by empirical evidence [40].

### 3.4. Variable Predisposition Theory

Variable Predisposition Theory, a cognitive approach developed to understand basic and acute suicide risk, was proposed by Rudd (2006) [41]. The theory focuses on the suicide risk process in addition to suicidal behaviour. While basic risk refers to the chronic or stable features of suicide risk over time, acute risk emphasises the dynamic features of suicide risk in response to external forces. In other words, while the basic risk is considered a ‘predisposition’, the acute risk is the activation of suicidal mode. While basic risk tends to be resistant to change over time, acute risk tends to be a reaction to situations such as fluctuations in mood and hopelessness, and changes in stress factors. Suicidal behaviour is the result of these two risk processes affecting each other. The Variable Predisposition Theory also provides a basis for explaining suicides that occur without any prepared plan [42,43].

The term mode was defined by Beck in 1996 as cognitive processes in which internal and external stimuli are interpreted. Modes, which constitute the structural networks of cognitive, emotional, motivational, physiological and behavioural schemes that are activated simultaneously with internal and external events, become active repeatedly, causing the next activation threshold to decrease. From this point of view, Rudd, Joiner and Dahm proposed a “suicide mode” consisting of cognitive, emotional, physiological and behavioural domains in 2001. The emergence and continuity of suicide are explained by the vulnerabilities that occur in these areas. If the individual has a sensitive suicidal belief system, recurrent crises will increase his/her vulnerability to future crises [16].

The cognitive domain includes problem-solving style, cognitive flexibility, cognitive rigidity and cognitive distortions. Examples include the belief that one is unloved, the belief of being a burden on others, the feeling of helplessness and the inability to tolerate distress. These distortions, called the ‘suicide belief system’, consist of two different predispositions, ‘cognitive rigidity’ and ‘emotion dysregulation’, and one of them is not less important than the other [32,41].

Cognitive rigidity can be defined as lacking cognitive flexibility. Cognitive flexibility is a person’s ability to cope with new and unexpected circumstances and, as a result, to be able to adapt their cognitive processes to those conditions is the ability to organise for the new process. An individual with cognitive flexibility resorts to new methods instead of trying to solve the problem by insistently repeating the methods that they have observed to be effective in previous conditions, but which are understood not to be effective in the new process. In cognitive rigidity, the individual cannot organise their cognitive skills in order to adapt to new and unexpected conditions. They try to solve the problem by persistently repeating the methods that do not contribute to adaptation to the new conditions that arise. Emotion dysregulation can be defined as a lack of awareness of emotional reactions, failure to understand emotional reactions, failure to accept emotional reactions, difficulty in controlling impulses when experiencing negative emotions and difficulty in goal-oriented behaviours when experiencing negative emotions.

The behavioural domain consists of a wide range of variables, including emotion regulation strategies and interpersonal interactions that are regulated to reduce emotional distress. The emotional domain consists of various emotional experiences (anger, guilt, depression, anxiety, etc.). The physiological domain includes physical and somatic experiences (autonomic arousal, pain and motor–sensory system activation, etc.) [44]. It is stated that this model makes a significant contribution to the evaluation of suicide cases by taking into account acute risk factors (current suicidal ideation, etc.) and basic (chronic) risk [16].

## 4. Conclusions

The theories considered first-generation suicide theories are insufficient to distinguish between suicidal thoughts and suicidal behaviour. These theories, which are based on clinicians’ individual experiences and clinical judgement, have integrated their perspectives on other human behaviours into suicidal behaviour. This may be reasonable as an argument for a theory, but suicide is the most complex of human behaviours. New approaches are needed to understand the intervening factors in the transformation of the survival instinct into suicidal behaviour or the transition from suicidal ideation to suicidal action. The theories developed since the beginning of the 2000s and named the second-generation suicide theories fulfil this need. Second-generation suicide theories argue that different factors play a role in suicidal ideation and suicide attempts or deaths due to suicide. In this sense, it can be said that second-generation suicide theories bring a new perspective to suicide. These theories have potential importance for the assessment of suicide risk, development of suicide prevention methods and planning and conducting research. All the theories have been tested to varying degrees and have important implications for the development of therapeutic and preventive interventions.

Suicide is a complex behaviour that needs to be addressed from a biopsychosocial perspective. While the concepts used in these theories have common points, there are also points where they differ. In the theories considered, risk factors, predisposing and protective factors are examined from many perspectives, including biological, psychological and sociological. Despite this multifaceted approach of the theories, the number of suicides is quite high in practice. It is recommended that future researchers examine whether this situation is due to the limited use of theoretical approaches in clinical practice or whether theoretical approaches need additional evidence for their biopsychosocial explanations.

## Data Availability

All information in the article is publicly available.
